# The brightest multi-colour phonon lasers

**DOI:** 10.1038/s41377-024-01648-2

**Published:** 2024-11-13

**Authors:** Mishkat Bhattacharya

**Affiliations:** 1 School of Physics and Astronomy, Rochester Institute of Technology, Rochester, NY USA; 2 Center for Coherence and Quantum Optics, University of Rochester, Rochester, NY USA

**Keywords:** Quantum optics, Nonlinear optics

## Abstract

A new device applies a single-colour electronic injection to create the brightest multi-colour phonon laser, with ten times more power and much narrower linewidth than others.

Phonons are the basic or quantized elements of mechanical vibrations. Phonon lasers, as mechanical analogues of optical lasers, are unique tools for not only fundamental studies of phononics but also diverse applications such as acoustic imaging and force sensing, etc. A phonon laser exhibits characteristics analogous to photonic lasers, including a clear threshold for transition from thermal to coherent oscillating motion, linewidth narrowing, and squeezing of phonon autocorrelations^1–3^. In other words, all phonons can be focused in a single lasing mode with the same frequency, moving in a highly consistent way, which is in sharp contrast to random distributions of thermal phonons. Compared to traditional sound waves, phonon lasers have superior coherence, which makes them an ideal tool to further enhance the accuracy of measurement. In high-precision biomedical sensing, for example, ordinary sound waves need to use pulse compression and other technologies to reduce the width of ultrasonic pulses. However, phonon lasers, with extremely narrow linewidth, can naturally provide better longitudinal resolution.

Substantial efforts have been devoted to realizing phonon lasers with ions, micro-resonators, membranes, semiconductor lattices, and photonic crystals. Notably, in 2023, Kuang and coworkers^4^ developed a strategy for the first time to achieve multi-colour phonon lasers by using an active levitated optical mechanical (LOM) system. Such levitated optomechanical devices, with minimal thermal noise and mechanical noise in high vacuum, allow flexible control of large-mass objects without relying on any internal discrete energy level structure. Besides, the optical gain of the active cavity compensates for the loss and lowers the power threshold. Subsequently, nonlinear mechanical harmonics emerge spontaneously above the lasing threshold. Such a device enables a wide range of applications, such as quantum phononics, multi-frequency mechanical sensors, and high-precision acoustic frequency combs. However, both the lasing strengths and the quality factors of the observed harmonics are typically very low, thus severely hindering their applications.

In the recent work published in eLight, Xiao et al. tackled this crucial issue and demonstrated a considerable improvement in several key features of multi-colour phonon lasers by the direct application of electronic injection locking into the active LOM system^5^. As shown in Fig. 1, the display system mainly consists of four sections: a dual-beam optical tweezer, a SiO_2_ microsphere, an active cavity and an electrode. The working principle is briefly summarized as follows. Firstly, the microsphere is trapped with the dual-beam optical tweezer. Secondly, the multi-colour phonon laser is excited by using the active cavity. The last but most important step is to apply a tunable alternating current field to the microsphere. By controlling the frequency of the current field, both the fundamental-mode and all higher-order harmonics of the phonon laser can be well locked, leading to giant enhancement of their qualities, including brightness, linewidths, frequency stabilities, and higher-order coherence. The brightness of the fundamental-mode phonon laser is enhanced by 3 orders of magnitude, compared to the work by Kuang et al., with also 5 orders linewidth narrowing. Giant enhancement can also be observed in the frequency stability, which leads to a longer trapping lifetime of the micro-object, i.e., from 1.3 min to over 1.2 h. Besides, the mechanical quality factor reaches 6.6 × 10^6^, which is the highest record for a microsphere phonon laser.

**Fig. 1:**
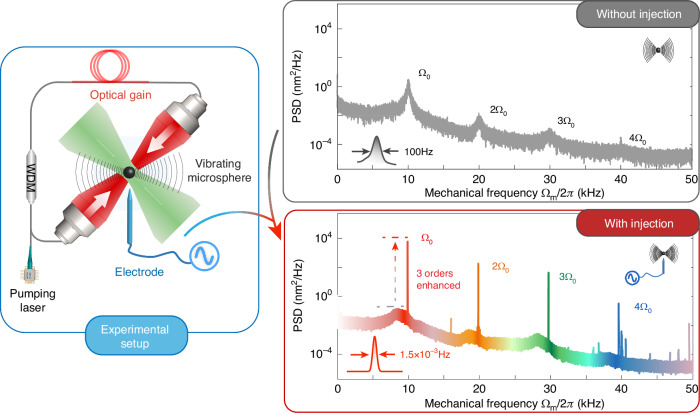
Experimental setup and results The method uses a single-colour electronic injection to remarkably enhance the performance of both the fundamental phonon laser mode and its high-order harmonics

Compared to the previous methods for improving the quality of phonon lasers, such as feedback control^3^, optical polarization control^6^, and Floquet engineering^7^, the approach used by Xiao et al. serves as a compelling example to improve the qualities of both the fundamental-mode phonon laser and all its higher-order harmonics for the first time. Although injection locking is a well-established technique^8–10^, new results reported in their work also include simultaneous locking of both the fundamental-mode phonon laser and all higher-order harmonics, giant improvements of nonlinear mechanical harmonics in the phonon-lasing regime, and clear evidence of the positive role of locking on enhancing higher-order coherence of phonon lasers. In all, this is a milestone work that drives phonon lasers towards practicality.

In summary, the marriage of injection locking and active gain, as demonstrated in this milestone work driving phonon lasers towards practicality, provides a novel and feasible solution to realize the giant enhancement of phonon lasers. Such effects are expected to be observed in other phonon laser systems using e.g., cold ions, vibrating membranes, and semiconductor lattices^1,2,11–13^. Besides, this hybrid electro-LOM system offers considerable promise as a platform for exploring novel applications of coherent electro-acoustic conversions, nonlinear phononics, and the development of coherent acoustic frequency combs. Moreover, such progress will provide basic guidance to other meaningful research, such as synchronization characteristics, collective many-body effects, and even entangled or squeezed phonon lasers.
